# Antioxidant and Inhibitory Activities of *Filipendula glaberrima* Leaf Constituents against HMG-CoA Reductase and Macrophage Foam Cell Formation

**DOI:** 10.3390/molecules29020354

**Published:** 2024-01-10

**Authors:** You Bin Cho, Hyunbeom Lee, Hui-Jeon Jeon, Jae Yeol Lee, Hyoung Ja Kim

**Affiliations:** 1 Center for Advanced Biomolecular Recognition, Korea Institute of Science and Technology, Seoul 02792, Republic of Korea; youbin1214@naver.com (Y.B.C.); hyunbeom@kist.re.kr (H.L.); 2 New Drug Development Center, Daegu-Gyeongbuk Medical Innovation Foundation, Daegu 41061, Republic of Korea; hjjeon@kmedihub.re.kr; 3 Department of Chemistry, College of Sciences, Kyung Hee University, Seoul 02447, Republic of Korea

**Keywords:** *Filipendula glaberrima*, antioxidant activity, 3-hydroxy-3-methylglutaryl-coenzyme A reductase (HMGR), foam cell formation, hydrolysable tannins, atherosclerosis

## Abstract

In our search for bioactive components, various chromatographic separations of the organic fractions from *Filipendula glaberrima* leaves led to the isolation of a new ellagitannin and a triterpenoid, along with 26 known compounds. The structures of the isolates were determined based on their spectroscopic properties and chemical evidence, which were then evaluated for their antioxidant activities, inhibitory activities on 3-hydroxy-3-methylglutaryl-coenzyme A reductase, and foam cell formation in THP-1 cells to prevent atherosclerosis. Rugosin B methyl ester (**1**) showed the best HMG-CoA reductase inhibition and significantly reduced ox-low-density lipoprotein-induced THP-1 macrophage-derived foam cell formation at 25 µM. In addition, no cytotoxicity was observed in THP-1 cells at 50 μg/mL of all extracts in the macrophage foam cell formation assay. Therefore, *F. glaberrima* extract containing **1** is promising in the development of dietary supplements due to its potential behavior as a novel source of nutrients for preventing and treating atherosclerosis.

## 1. Introduction

Conventional medicinal plants consist of abundant phytochemicals, encompassing phenolic compounds (such as quinones, tannins, coumarins, flavonoids, phenolic acids, lignans, and stilbenes), terpenoids (including carotenoids), nitrogen compounds (alkaloids, betalains, and amines), and various other naturally occurring metabolites known for their strong antioxidant properties. Thus, these phytochemicals are ideal ingredients for inclusion in functional foods [1,2].

Phytochemicals have attracted considerable attention as sources of functional ingredients in food formulations because of their numerous health benefits. In light of the growing demand for functional foods aimed at promoting a healthy lifestyle, there is a need for novel sources of raw materials that can offer consumers both desirable taste and beneficial functionality. The beneficial effects of these plant constituents have been partly linked to the abundance of various polyphenolic compounds, which exhibit antioxidant activity and/or free radical-scavenging properties in vitro [3]. Polyphenols and tannins have gained significant attention due to their impact on the odor, flavor, and color of beverages and foods and health-promoting properties [4,5]. The efficacy and safety of dietary supplements and herbal medicines sourced from purified natural compounds or extracted from edible plants have contributed to their widespread popularity [6].

Cholesterol is present in the human body and is an essential substance involved in various biochemical reactions as a component of the cell membrane. However, excess cholesterol may accumulate in vascular endothelial cells or the endothelium, causing vascular diseases, such as hyperlipidemia, and secondary diseases, such as arteriosclerosis, hypertension, obesity, and diabetes. Atherosclerosis is a chronic inflammatory process involving hypercholesterolemia, low-density lipoprotein (LDL) oxidation, hypertension, and platelet aggregation and is initiated by the accumulation of macrophage foam cells in the subendothelial arterial space [7,8].

Macrophages, integral cellular constituents of the host defense system, perform vital functions in both innate and adaptive immunity. Certain actions of macrophages can be beneficial, such as eliminating oxidized lipoproteins and facilitating the release of cholesterol derived from lipoproteins to high-density lipoprotein receptors, aiding in reverse cholesterol transport [9]. Many cholesterol-lowering agents, including nicotinic acid, plant sterols, and statins, have been introduced for clinical use [10]. Statins, such as pravastatin and lovastatin, decrease serum cholesterol levels by inhibiting hepatic 3-hydroxy-3-methylglutaryl-coenzyme A (HMG-CoA) reductase, the rate-limiting enzyme in cholesterol biosynthesis. HMG-CoA reductase is an enzyme that mediates the synthesis of mevalonic acid, an intermediate in the biosynthetic pathway of sterol or isoprenoid compounds. When the activity of HMG-CoA reductase is lowered, the lipid and cholesterol levels in the blood can be lowered by inhibiting cholesterol [10]. 

The oxidation of LDL potentially plays a significant role in the progression of atherosclerosis [11]. Oxidative stress serves as a prominent risk factor in the oxidation of LDL. The internalization of oxidized LDL (ox-LDL) by endothelial cells and macrophages contributes to endothelial dysfunction and the formation of foam cells. Foam cells are a major cause of atherosclerosis, while increased blood cholesterol levels are one of the most critical risk factors for the pathogenesis of coronary heart disease [7]. Therefore, lowering LDL oxidation is a useful strategy to prevent atherogenic diseases, for which plant-derived dietary components can play an important role.

*Filipendula glaberrima* Nakai (Rosaceae), also known as Korean meadowsweet, is a perennial plant found in the northern mountains of Central Korea. Its young leaves are wild herbs that are blanched and eaten as vegetables. *F. glaberrima* exhibits a notable abundance of phenolic compounds, including tannins and flavonoids, making it suitable for medicinal applications such as alleviating spasms, inducing sedation, addressing neuralgia and gout, providing analgesic relief, managing arthritis, and exerting anti-inflammatory effects [12]. Therefore, this study reports the isolation and structural elucidation of the corresponding functional ingredients of *F. glaberrima*, the active constituents, and their biological effects on antioxidant activity and HMG-CoA reductase (HMGR) inhibition. Furthermore, the inhibitory effects on macrophage foam cell formation in THP-1 cells were assessed for two newly discovered compounds in conjunction with the organic extracts. These findings hold promise for the future development of valuable functional ingredients for nutraceuticals.

Additionally, two new compounds, together with the organic extracts, were evaluated for their inhibitory activities on macrophage foam cell formation in THP-1 cells. The obtained results are expected to play a role in the development of valuable functional ingredients for nutraceuticals.

## 2. Results and Discussion 

### 2.1. Structure Elucidation

**Figure 1:**
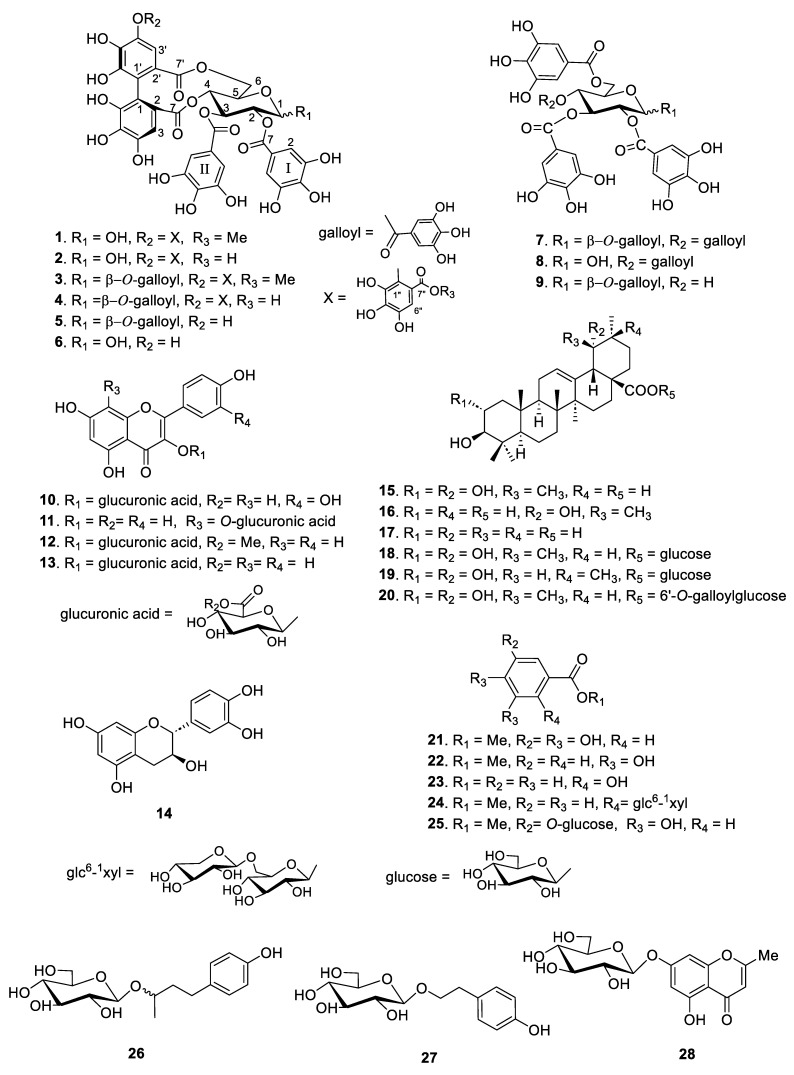
Structures of compounds **1**–**28** isolated from the leaves of *F*. *glaberrima*.

Based on the structural differences between the isolated compounds, they were divided into four types. Compounds **1**–**9** are hydrolyzable tannins, compounds **10**–**14** are flavonoid glycosides, compounds **15**–**20** are triterpenes, and compounds **21**–**28** are phenolic compounds (Figure 1). Among them, compounds **1** and **20** were identified as new compounds. The structural determination procedures for compounds **1** and **20** are described below:

Rugosin B methyl ester (**1**) was obtained as a brownish amorphous powder with a molecular formula of C_42_H_32_O_27_ as deduced from the HR-ESI-MS (Figure S1) measurement at *m*/*z* 967.1053 [M − H]^−^. The ^1^H and ^13^C NMR spectra of **1** indicated that the tannin formed an anomeric mixture (α-anomer: β-anomer = 4:3), which is a structural feature similar to that of rugosin B (**2**), except for the presence of a methyl ester signal at δ_H_ 3.74 and δ_C_ 52.5 in the valoneoyl moiety. The ^1^H NMR spectrum of **1** (Figure S2) exhibited additional methyl ester signals at δ_H_ 3.75 (β-anomer) and 3.74 (α-anomer), belonging to the ester of the valoneoyl group. The two proton singlet signals at δ_H_ 7.04 and 6.95 and three singlet signals at δ_H_ 7.06, 6.52, and 6.20 were assigned to two galloyl groups and a valoneoyl group in the α-anomer signals, respectively. Furthermore, the presence of two galloyl groups (δ_H_ 7.02 and 6.91) and a valoneoyl group (δ_H_ 6.95, 6.48, and 6.19) was supported by the β-anomer signals in the ^1^H NMR spectrum (Table 1). 

The ^13^C NMR spectrum (Figure S3) indicated signals corresponding to two sets of four carbon signals and two carbonyl carbons for two symmetrical galloyl groups and a valoneoyl group from α- and β-anomers, respectively (Table 1). Based on the ^1^H-^1^H COSY and HMQC spectral data (Figures S4 and S5), the proton signals of the methyl ester moiety in **1** were assigned. In the HMBC experiment (Figures S6 and S7), the protons at δ_H_ 5.11 (H_glc_-2α) and 7.04 (H_gal-I_-2 and 6) were crossed with the signal at δ_C_ 167.6 (C_gal-I_-7), which showed a cross-peak between the protons at δ_H_ 5.82 (H_glc_-3α) and 6.95 (H_gal-II_-2 and 6) and the carbon signal at δ_C_ 167.9 (C_gal-II_-7), indicating that the hydroxyl groups on glucose C-2 and -3 were acylated by galloyl groups. The HMBC correlations demonstrated the presence of connections of H_glc_-4 (δ_H_ 5.09) and H-3 (δ_H_ 6.52) with C-7 of the valoneoyl group (δ_C_ 169.2). Additionally, we observed correlations of H_glc_-6 (δ_H_ 5.24 and 3.76) and H-3′ (δ_H_ 6.20) with C-7′ of the valoneoyl group (δ_C_ 169.4). These findings indicate that the hexahydroxydiphenoyl component was positioned at O-4 and -6 of the glucose moiety, while the orientation of the valoneoyl group matched that of rugosin B. Moreover, the methoxy group located at δ_H_ 3.74 and the H-6″ signal at δ_H_ 7.06 displayed correlations with the carbonyl group (C-7″) at δ_C_ 167.5 within the galloyl portion of the valoneoyl group. This correlation suggests that the carboxyl group of the galloyl component formed a methyl ester. In a previous study, methyl esters of rugosin B were synthesized as artefacts when prostratin B was treated with a mixture of MeOH and hot water [13]. To test whether compound **1** is an artefact in this study, rugosin B in MeOH solution was kept at room temperature for 3 d in the presence of Sephadex LH-20, which is a condition similar to that of the isolation procedure. Rugosin B did not change with any formulation, indicating that compound **1** was not an artefact formed during the isolation process. Therefore, this finding confirmed that compound **1** is a new natural compound, a rugosin B methyl ester.

6′-*O*-Galloylrosamultin (**20**) was obtained as an amorphous powder, and its molecular formula C_43_H_62_O_14_ was determined by HR-ESI-MS (Figure S8) which showed a molecular ion peak [M − H]^−^ at *m*/*z* 801.4069 (calculated for C_43_H_61_O_14_, 801.4061). The molecular formula mentioned above was validated through the analysis of the ^13^C NMR spectroscopic data. The ^1^H and ^13^C NMR spectra (Figures S9 and S10) of compound **20** revealed a structural characteristic resembling that of rosamultin, which is derived from *Rosa multiflora* [14], with the exception of the inclusion of a galloyl group at the C-6′-hydroxyl position of glucose. The ^1^H NMR spectrum of **20** exhibited an additional singlet signal at δ_H_ 7.09, corresponding to the aromatic protons in the galloyl moiety. The presence of this signal was additionally corroborated by the correlations observed between the methylene peaks at δ_H_ 4.40 and 4.33 of glucose (C-6′) and the carbonyl peak at δ_C_ 168.6 (C-7″) of the galloyl group, as evidenced in the ^1^H-^1^H COSY, HMQC and HMBC spectra (Figures S11–S13). Thus, the structure of **20** was established as 6′-*O*-galloylrosamultin.

The spectral data of the other known compounds were consistent with those reported in previous studies as follows: rugosin B (**2**) [15], rugosin A methyl ester (**3**) [16], rugosin A (**4**) [15], eugeniin (**5**), tellimagrandin I (**6**) [17], 1,2,3,4,6-penta-*O*-galloyl-β-d-glucose (**7**) [18], 2,3,4,6-tetra-*O*-galloyl-d-glucose (**8**), 1,2,3,6-tetra-*O*-galloyl-*β*-d-glucose (**9**) [19], quercetin 3-glucuronic acid (**10**) [20], kaempferol 8-*O*-glucuronic acid (**11**), kaempferol 3-glucuronic methyl ester (**12**), kaempferol 3-glucuronic acid (**13**) [21], catechin (**14**) [22], tomentic acid (**15**), benthamic acid (**16**) [23], ursolic acid (**17**) [24], rosamutin (**18**) [25], arjunetin (**19**) [26], methylgallate (**21**), methylprotocatechuate (**22**), salicylic acid (**23**) [27], gaultherin (**24**) [28], methyl gallate 3-*O*-β-d-glucopyranoside (**25**), (*R*/*S*)-rhododendrin (**26**) [29], salidroside (**27**) [30], and undulatoside A (**28**) [31] as shown in Figure 1. In addition, 26 known compounds (**2**–**19** and **21**–**27**) were identified by comparing their physical and spectroscopic data with those reported in the mentioned literature, respectively.

### 2.2. Antioxidant Capacity 

The DPPH and superoxide anion radical-scavenging activities are frequently utilized to evaluate the antiradical/antioxidant capability of isolated compounds for comparison with those of various reference standards. As shown in Table 2, the findings of DPPH radical scavenging revealed that the ethyl acetate and butanol fractions of *F. glaberrima* had the highest antioxidant activities with an IC_50_ value of 4.62 and 5.25 μg/mL, respectively. Additionally, the ethyl acetate and butanol fractions had strong scavenging activities against the superoxide anion radical based on their low IC_50_ values (4.07 and 4.64 μg/mL, respectively), indicating better activities than quercetin (IC_50_ of 21.3 μM). The recorded IC_50_ value of the methanol extract was lower than that of the standard, with a strong antioxidant potential near the standard.

Among the active compounds, compounds **1**–**6** containing hexahydroxydiphenoyl (HHDP) or valoneoyl groups are a class of ellagitannins, and compounds **7**–**9** with only galloyl groups are a class of gallotannins (Figure 1). Rugosin B methyl ester (**1**) showed strong scavenging activity with an IC_50_ value of 3.62 µM, which was 8–11-fold more potent than quercetin (IC_50_ of 42.1 µM), ascorbic acid (IC_50_ of 31.7 µM), and Trolox (IC_50_ of 31.3 µM) and 4.7-fold more potent than quercetin (IC_50_ of 17.3 μM). Compounds **2**–**9** exhibited substantial DPPH radical-scavenging activities with an IC_50_ range of 3.19–4.70 µM, which was better than the positive controls (Table 3).

A comparison of ellagitannins **1**–**9** revealed that their radical-scavenging activities were significantly enhanced by increasing the number of galloyl units or HHDP moieties, including a valoneoyl group in the compounds, and the highest radical-scavenging activity was particularly observed for rugosin A (**4**) (IC_50_ of 3.19 and 3.21 μM against DPPH and superoxide anion radical, respectively). In addition, the organic fractions and isolated compounds from *F. glaberrima* were tested for their lipid peroxidation activities (Table 3). Among the fractions, the ethyl acetate fraction showed the most potent inhibitory effect, with an IC_50_ value of 9.67 μg/mL, followed by the butyl alcohol fraction (IC_50_ = 18.8 μg/mL) and methanol extract (IC_50_ = 26.3 μg/mL). Ethanolic extracts (30% and 70% ethanol extracts) exhibited moderate inhibitory effects with an IC_50_ of 40.1 and 34.7 μg/mL, respectively (Table 2). Among the isolates, hydrolyzable tannins **1**–**9** showed prominent inhibitory effects with an IC_50_ range of 3.54–5.92 μM. Quercetin 3-glucuronic acid (**10**) showed moderate inhibition of lipid peroxidation activity (IC_50_ of 34.1 μM), comparable to that of Trolox (IC_50_ of 33.2 μM), which was used as a positive control. Flavonols and hydrolyzable tannins containing multiple adjacent OH groups, particularly catechol groups, exhibited enhanced radical-scavenging activities against DPPH [32]. It has been reported that glycosidation at C3 of the C-ring of flavonols leads to a decrease in their antioxidant activity [33], which aligns with the findings observed for compounds **11**–**13** (all derivatives of kaempferol glycosides) (Figure 1). These experimental data are in good agreement with the literature [32,33].

### 2.3. Inhibitory Effect of HMG-CoA Reductase

The methanol extract from the *F. glaberrima* leaves was sequentially fractionated with dichloromethane, ethyl acetate, and butanol. In Table 2, the butyl alcohol fraction had the highest HMGR inhibitory activity, with an IC_50_ value of 0.74 μg/mL, followed by the ethyl acetate fraction (IC_50_ = 1.73 μg/mL) and methanol extract (IC_50_ = 2.86 μg/mL). 

**Figure 2:**
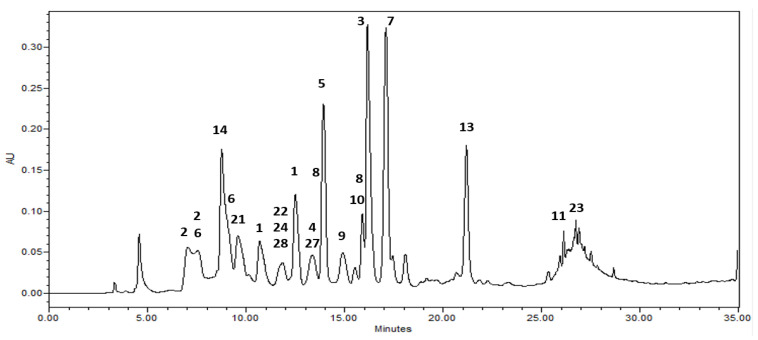
HPLC chromatograms of the ethyl acetate fraction from leaves of Korean meadowsweet (*Filipendula glaberrrima*). The detection wavelength was 254 nm. **1**: rugosin B methyl ester, **2**: rugosin B, **3**: rugosin A methyl ester, **4**: rugosin A, **5**: eugeniin, **6**: tellimagrandin I, **7**: 1,2,3,4,6-penta-*O*-galloyl-β-d-glucose, **8**: 2,3,4,6-tetragalloyl glucose, **9**: 1,2,3,6-tetra-*O*-galloyl-*β*-d-glucose, **10**: quercetin 3-glucuronic acid, **11**: kaempferol 8-*O*-glucuronic acid, **13**: kaempferol 3-glucuronic acid, **14**: catechin, **21**: methylgallate, **22**: methylprotocatechuate, **23**: salicylic acid, **24**: gaultherin, **27**: salidroside, **28**: undulatoside A.

Among the isolates, rugosin B methyl ester (**1**) showed the highest inhibitory activity against the HMGR enzyme with an IC_50_ value of 1.46 μM, the structure of which is different from that of rugosin A methyl ester (**3**) (IC_50_ = 8.40 μM) with respect to the presence of the galloyl group at the anomeric position of rugosin B methyl ester (**1**). In addition, 1,2,3.4.6-penta-*O*-galloyl-β-d-glucoside (**7**) showed reasonable inhibitory activity with an IC_50_ value of 4.98 μg/mL, whereas an analog of **7**, 2,3.4.6-tetra-*O*-galloyl-d-glucoside (**8**) exhibited three times less activity (IC_50_ = 13.8) than **7** (Table 3). Moreover, 1,2,3,6-tetra-*O*-galloyl-β-d-glucoside (**9**), without a galloyl unit at the C-4 position, resulted in a weak effect, indicating that the position of the galloyl unit on the C4 core could play an important role in the inhibition of HMGR, even for compounds with the same number of galloyl moieties. The analytical HPLC chromatogram (Figure 2 and Figure S14) of the ethyl acetate fraction in this study showed relatively high contents of rugosin A methyl ester (**3**) and 1,2,3,4,6-penta-*O*-galloyl-β-d-glucoside (**7**). 

Hence, the notable inhibitory impact of *F. glaberrima* leaf extract on HMGR may be attributed to its significant ellagitannin content. However, to establish a clearer understanding of the connection between HMGR inhibitory activity and the structures of hydrolyzable tannins, further comprehensive analysis utilizing ellagitannin derivatives is warranted.

### 2.4. Inhibitory Effect of Foam Cell Formation in THP-1 Cells 

**Figure 3:**
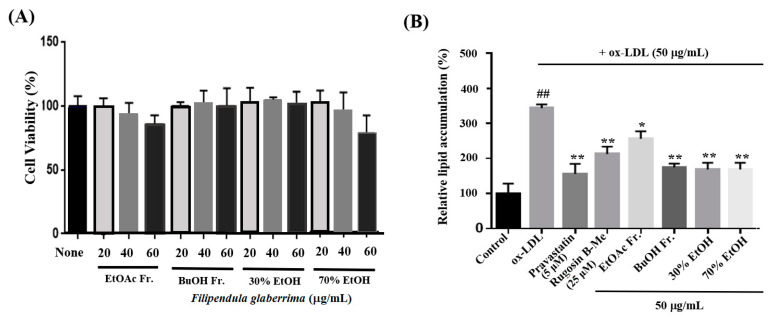

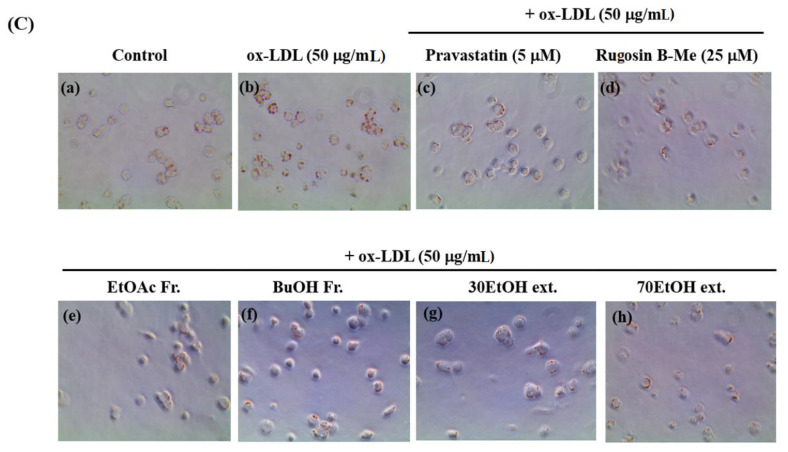
Effects on lipid accumulation in THP-1 macrophage foam cells. THP-1 macrophages were treated with 50 μg/mL ox-LDL alone or in combination with various samples from the leaves of *F. glaberrima* and pravastatin as a positive control. (**A**) Cells were treated with gradient dilutions of organic extracts (20–60 μg/mL) and subjected to cell viability experiments using the MTT method. (**B**) Quantitative analysis of intracellular lipid contents based on the absorbance of intracellular dye extracted with isopropanol at 450 nm. The results are reported as the means ± SD from three independent experiments performed in triplicate. ^##^ *p* < 0.01 compared with control; * *p* < 0.05 and ** *p* < 0.01 compared with treatment with ox-LDL alone. (**C**) Representative optical microscopy images of Oil Red O-stained lipid droplets at a magnification of ×40. Cells in the (**a**) control, (**b**) ox-LDL alone, (**c**) pravastatin (5 μM), (**d**) rugosin B methyl ester (25 μM), and 50 μg/mL concentration of (**e**) ethyl acetate fraction, (**f**) butanol fraction, (**g**) 30% ethanol extract and (**h**) 70% ethanol extract. Control cells were cultured for the same time without any treatments.

The uptake of ox-LDL by macrophages is a critical factor in the formation of foam cells and the development of atherosclerosis. THP-1 cells have been widely employed as a convenient cellular model for investigating foam cell formation and studying macrophage behavior in vitro. THP-1 macrophages were exposed to 50 μg/mL of ox-LDL, with or without the inclusion of solvent extracts, two novel compounds (**1** and **20**), and pravastatin (used as a positive control), for 16 h. This approach aimed to examine the impact of these substances on lipid accumulation and the formation of foam cells. First, we observed the effects of different concentrations (20, 40, and 60 μg/mL) of the solvent extracts on the THP-1 macrophage activity. While higher concentrations of the samples resulted in reduced viability of THP-1 macrophages, a maximum concentration of 50 μg/mL did not have any adverse effects on cell viability (Figure 3A). As a result, we selected and optimized the concentration of 50 μg/mL for the solvent extract to be used in the subsequent experiments. According to the findings presented in Figure 3B,C, the administration of ethanolic and organic solvent extracts resulted in a notable decrease in lipid accumulation within THP-1 macrophage foam cells when compared to the group treated with ox-LDL alone (model group). Following the extraction of the intracellular dye using isopropanol, a quantitative assessment of the intracellular lipid content was conducted. The ethanolic extracts and butanol fraction exhibited a reduction in lipid content within THP-1 macrophage foam cells, reaching approximately 70–72% at a concentration of 50 μg/mL. This reduction was significantly different from the lipid content of cells treated solely with ox-LDL. The observed results are comparable to the 77% inhibitory effect of pravastatin, which was used as a positive control at a concentration of 5 μM. The ethyl acetate fraction also showed an inhibitory effect on foam cell formation of 36% at a concentration of 50 μg/mL compared to the model group. Moreover, at a safer concentration of 25 µM of compound **1**, both the number of intracellular red-stained particles and the clustering of foam cells were lower (54%) than those in the model group. The results showed that compound **1** significantly reduced lipid accumulation. 

Previous studies on the chemical composition of *Filipendula* species have revealed substantial quantities of polyphenolic compounds, with particular emphasis on notable constituents such as flavonol glycosides and ellagitannins [34]. These plants are worthy of further extensive investigation in phytochemistry and in terms of their health-promoting effects. Furthermore, many studies have indicated that dietary antioxidant supplements and food plants, which are mainly rich in tannins and polyphenols, play a major role in controlling or preventing various diseases, such as cardiovascular diseases, diabetes, and even cancer, by slowing or reducing the level of oxidative stress, principally by protecting lipoproteins from lipid peroxidation without any obvious toxicity.

## 3. Materials and Methods

### 3.1. General Experimental Procedures 

High-resolution electrospray ionization mass spectrometry (HR-ESI-MS) data were obtained using an Agilent 6210 ESI/TOF mass spectrometer (Agilent Technologies, Santa Clara, CA, USA). Semi-preparative chromatography was performed on a Waters 1525 pump and a 2996 photodiode detector equipped with a Luna C18 column (5 μm, 250 × 10 mm, Phenomenex, Torrance, CA, USA) and used for analytical high-performance liquid chromatography (HPLC; Waters Corporation, Milford, MA, USA) with a Luna C18 column (5 μm, 250 × 4.6 mm). ^1^H and ^13^C-nuclear magnetic resonance (NMR) spectra were recorded on a Bruker spectrometer (Bruker BioSpin GmbH, Rheinstetten, Germany) at 400, 600, and 800 MHz for ^1^H and 100, 150, and 200 MHz for ^13^C. 

Enzyme assays were performed using an Epoch Microplate spectrophotometer (BioTek Instruments, Inc., Winooski, VT, USA) in transparent 96-well plates (Greiner Bio-One, Kremsmünster, Austria). All other reagents and chemicals were purchased from Sigma-Aldrich (Saint Louis, MO, USA) and different commercial suppliers and were of analytical grade.

### 3.2. Plant Material 

The leaves of *F. glaberrima* were collected from a wide-growing habitat in Yanggu County, Gangwon Province, Republic of Korea, in May 2018 and verified by Professor Emeritus, Chang-Soo Yook (Department of Pharmacognosy, Kyung Hee University). A voucher specimen (308-43A) was deposited in the herbarium of the Korea Institute of Science and Technology (KIST) in Seoul, Republic of Korea.

### 3.3. Extraction and Isolation 

To obtain the combined MeOH-soluble extract, air-dried leaves of *F. glaberrima* (840 g) were powdered and subjected to room temperature extraction with MeOH (6 L × 3) for a duration of 5–6 days. The resulting extract was concentrated under a vacuum, yielding a residue (194.6 g). An aliquot of this residue (61.4 g) was suspended in H_2_O (600 mL) and partitioned with CH_2_Cl_2_ (600 mL × 3), EtOAc (600 mL × 3), and *n*-BuOH (600 mL × 3). The EtOAc fraction (12.5 g) was subjected to Sephadex LH-20 CC and eluted with MeOH to yield 11 fractions (EA–EK). Compound **15** (14.0 mg), compound **16** (70.1 mg), and compound **17** (28.0 mg) were obtained through the purification of the insoluble solid (914 mg) from fraction EC. This purification process involved dissolving fraction EC (4.1 g) in methanol, followed by RP-18 column chromatography using a solvent system of CH_3_CN-H_2_O (70:30 → 80:20). The filtrate of fraction EC was subjected to RP-18 CC using a mixture of CH_3_CN–H_2_O (65:35 → 95:5) solvent system to yield 11 fractions (EC1–EC11). Fraction EC2 (460.2 mg) was further purified by RP-18 CC using gradient elution with MeOH–H_2_O (40 → 70%) to yield compounds **15** (7.6 mg), **18** (20.0 mg), **19** (1.6 mg), **20** (8.7 mg), and 12 fractions (EC2a–EC2l). Fraction EC2i was subjected to RP-18 CC (45 → 70% MeOH) to yield compounds **21** (86.8 mg), **22** (81.1 mg), and **23** (12.7 mg). Fraction EK (1.18 g) was chromatographed on an RP-18 CC using gradient elution with MeOH–H_2_O (30 → 80%, *v*/*v*) to obtain 12 subfractions (EK1–EK12). EK7 (916.6 mg) was subjected to Sephadex LH-20 (MeOH) to yield compounds **3** (347.1 mg), **4** (75.2 mg), **5** (342.3 mg), and **7** (47.7 mg). Fraction EK (792.9 mg) was subjected to RP-18 CC by gradient elution with MeOH–H_2_O (40% to 80%, *v*/*v*), and eight subfractions (EK1–EK8) were obtained according to their TLC profiles. Fraction EK2 (543.7 mg) was subjected to Toyopearl HW-40 CC and further separated repeatedly using semi-preparative HPLC by gradient elution with CH_3_CN–H_2_O (15 into 40%, *v*/*v*) to afford compounds **1** (35.4 mg) and **6** (235.1 mg). Compounds **2** (14.0 mg), **8** (7.9 mg), and **9** (8.3 mg) were obtained from EK2a using semi-preparative HPLC with a gradient elution of CH_3_CN (15 into 40%, *v*/*v*) in H_2_O.

By subjecting the BuOH fraction (10.1 g) to Sephadex LH-20 CC and eluting with MeOH, a total of 15 fractions (B1–B15) were obtained. Fraction B4 (576.6 mg) was further subjected to Sephadex LH-20 CC and eluted with MeOH, resulting in the isolation of five subfractions (B4A–B4E). Fraction B4B (85.2 mg) was chromatographed to RP-18 by gradient elution with MeOH–H_2_O (50 into 90%, *v*/*v*) to obtain compounds **10** (15.9 mg), **12** (1.54 mg), **13** (53.3 mg), and **14** (5.3 mg). Fraction B5 (344.7 mg) was subjected to Sephadex LH-20 CC, eluted with 70% MeOH, and further separated repeatedly with RP-18 CC by gradient elution with MeOH–H_2_O (50 → 70%, *v*/*v*) to yield compound **11** (6.3 mg). Fraction B2 (2.07 g) was subjected to Sephadex LH-20 CC and eluted with 70% MeOH to give ten fractions (B2A–B2J). Fraction B2H (149.5 mg) was applied to a silica gel column using a mixed solvent of CH_2_Cl_2_–MeOH–H_2_O (5:1:0.1) to afford compounds **24** (124.9 mg), **25** (7.3 mg), and **26** (4.0 mg). Fraction B2C (16 mg) was subjected to preparative RP-18 TLC (MeOH–H_2_O, 40:60) to yield compounds **27** (4.8 mg) and **28** (5.0 mg).

**Rugosin B methyl ester (1)**: brownish amorphous powder; HR-ESI-MS *m*/*z* 967.1053 [M − H]^−^ (calculated for C_42_H_31_O_27_, 967.1058). ^1^H NMR (CD_3_OD, 400 MHz) and ^13^C NMR (CD_3_OD, 100 MHz) spectra are shown in Table 1.

**6’-O-Galloylrosamultin (20)**: amorphous powder; HR-ESI-MS *m*/*z* 801.4069 [M − H]^−^ (calculated for C_43_H_61_O_14_, 801.4061). ^1^H NMR (CD_3_OD, 400 MHz) δ (ppm): 0.67, 0.76, 0.98, 1.19, 1.29 (each 3H, 6 × CH_3_), 0.93 (3H, d, *J* = 6.8 Hz), 2.57 (1H, s, H-18), 2.88 (1H, d, *J* = 9.6 Hz, H-3), 3.36–3.68 (3H, m, H-2′,3′,4′), 3.57 (1H, m, H-2), 3.67 (1H, m, H-5′), 4.33 (1H, dd, *J* = 5.6, 12.0 Hz, H-6′), 4.40 (1H, dd, *J* = 2.0, 12.0 Hz, H-6′), 5.29 (1H, br s, H-12), 5.42 (1H, d, *J* = 8.0 Hz, H-1′), 7.09 (2H, s, H-2″, 6″); ^13^C NMR (CD_3_OD, 100 MHz) δ (ppm): 16.6 (C-30), 16.9 (C-25), 17.4 (C-24), 17.9 (C-26), 19.4 (C-6), 24.7 (C-11), 24.8 (C-27), 26.4 (C-16), 26.9 (C-29), 27.1 (C-21), 29.3 (C-15, 23), 33.9 (C-7), 38.4 (C-22), 38.9 (C-10), 40.4 (C-4), 41.2 (C-8), 42.4 (C-14), 42.6 (C-20), 48.0 (C-1), 48.4 (C-9, overlapped with solvent), 49.6 (C-17), 54.6 (C-18), 56.6 (C-5), 65.1 (C-6′), 69.5 (C-2), 71.5 (C-4′), 73.7 (C-2′), 73.8 (C-19), 75.8 (C-5′), 78.1 (C-3′), 95.5 (C-1′), 110.4 (C-2″, 6″), 121.3 (C-1″), 129.7 (C-12), 139.5 (C-4″), 139.8 (C-13), 146.4 (C-3″, 5″), 168.6 (C-7″), 178.9 (C-28).

### 3.4. Measurement of Antioxidant Activity 

To assess the antioxidant activity of the isolated compounds and organic extracts, various assays including 2,2-diphenylpicrylhydrazyl (DPPH) [35], superoxide anion radical scavenging [36], and lipid peroxidation [37] were conducted (see the Supporting Information). Lipid peroxidation was carried out following the guidelines for the handling and utilization of laboratory animals and received approval from the Animal Research Ethics Committee of the Korea Institute of Science and Technology (approval number: KISTIACUC-2018-081). As positive controls, ascorbic acid, quercetin, resveratrol, and Trolox were employed. Triplicate measurements were performed for each sample, and the mean values were determined. The results are presented as the mean ± standard deviation (SD).

### 3.5. HMG-CoA Reductase (HMGR) Inhibition Assay 

The HMGR activity assay was optimized using a 96-well microplate reader to measure nicotinamide adenine dinucleotide phosphate (NADPH) oxidation during enzyme turnover. Each well was loaded with a mixture comprising 89 μL of 50 mM sodium phosphate buffer (pH 6.8), 0.8 mM NADPH, and 2 μg of the enzyme (2–8 units/mg). To initiate the reaction, 1 μL of the test sample and 10 μL of 0.8 mM HMG-CoA were introduced to the well. As a positive control, 10 μM pravastatin was utilized, while blank DMSO served as the negative control. The activity of HMGR was assessed by monitoring the reduction in NADPH absorbance at 340 nm for a duration of 900 s at 37 °C.

### 3.6. Assay for Foam Cells Formation in THP-1 Cells 

#### 3.6.1. Cell Culture

THP-1 cells (ATCC, TIB-202, Manassas, VA, USA) were obtained and maintained in RPMI-1640 medium (Cytiva, UT, USA), supplemented with 10% fetal bovine serum (FBS; Hyclone, Cytiva, Logan, UT, USA), penicillin (100 U/mL), streptomycin (100 μg/mL), and 2-mercaptoethanol (Sigma-Aldrich, Saint Louis, MO, USA) at a final concentration of 0.05 mM. The cells were cultured at 37 °C in a 5% CO_2_ atmosphere with humidity. The THP-1 cells in the suspension were treated with 50 nM phorbol-12-myristate-13-acetate (Sigma-Aldrich, USA) for 48 h. When the cells were attached to the plate, the medium was replaced with 1% FBS, and the starvation condition was maintained for 6 h. The cells were pretreated with pravastatin (Sigma-Aldrich, USA) and the extracts for 2 h. Ox-LDL (Invitrogen, Carlsbad, CA, USA) was treated with 50 μg/mL to form a foam cell and allowed to stand for a further 16 h.

#### 3.6.2. Oil Red O Staining 

THP-1 cells were seeded in 24-well plates at a density of 5 × 10^5^ cells/mL and pretreated as described above. The cells were fixed with 4% paraformaldehyde (Biosesang, Gyeonggi, Republic of Korea) and washed thrice with cold phosphate-buffered saline (Cytiva, Logan, UT, USA). Oil Red O (Sigma-Aldrich, USA) was diluted in isopropanol and filtered to prepare a solution. The fixed THP-1 cells were treated with Oil Red O solution for 10 min and then washed four times with distilled water. The cells were sequentially imaged using an optical microscope, eluted with 100% isopropanol, and transferred to a 96-well plate. The absorbance at 450 nm was measured using a multi-plate reader (Synergy Neo, BioTek Instruments, Inc., Winooski, VT, USA) and statistically processed.

### 3.7. Statistical Analysis 

The results presented are the mean ± SD derived from a minimum of three separate experiments, and statistical significance was assessed using an unpaired Student’s *t*-test via GraphPad Prism (version 6, GraphPad Software, CA, USA). *p* values lower than 0.05 were regarded as statistically significant.

## 4. Conclusions

Atherosclerosis is connected with profound disruptions in cholesterol metabolism. Herbs have been widely used for medicinal purposes up to the present day, despite recent efforts to understand their therapeutic effects and mechanisms. The mechanisms of herbal medicines are diverse, such as lowering blood lipid levels, anti-oxidative effects, and inhibition of plaque formation. 

Our results suggest proceeding with further investigations, although derived from limited studies of plant extracts with anti-atherosclerosis activity, show that *F. glaberrima* leaves possess potent antioxidant and inhibitory activities against HMGR and foam cell formation.

Hence, the potential of *F. glaberrima* to inhibit HMGR can be regarded as a cholesterol-lowering strategy, which could potentially decrease the likelihood of atherosclerosis development. Nonetheless, further comprehensive investigations utilizing an animal model of atherosclerosis are required to ascertain its efficacy and establish its potential implications in the prevention and treatment of atherosclerosis.

## Figures and Tables

**Table 1:** ^1^H and ^13^C NMR data of **1** (400 MHz for ^1^H, 100 MHz for ^13^C, CD3OD) ^a^.

Position	δ_H_		δ_C_
	α-anomer	β-anomer	α-/β-anomer
glucose-1	5.47, d (3.6)	5.06, d (7.6)	91.7/97.0
2	5.11, dd (3.6, 10.0)	5.18, dd (7.6, 9.6)	73.4/74.3
3	5.82, t (10.0)	5.58, t (9.6)	72.0/73.9
4	5.09, t (10.0)	5.09, t (10.0)	71.9/71.7
5	4.61, br dd (7.2, 9.6)	4.17, dd (6.8, 9.6)	67.5/72.7
6	5.24, br dd (6.8, 13.6)	5.33, br dd (6.0, 13.6)	64.3/64.2
	3.76, br d (13.6)	3.84, br d (13.2)	
galloyl-1			120.8, 120.6/120.9, 120.7
2/6	7.04, 6.95	7.02, 6.91	110.4, 110.38, 110.5/110.33
3/5			146.38, 146.21/146.35, 146.19
4			140.14, 139.94/140.0, 139.97
7			167.9, 167.6/167.7, 167.1
valoneoyl-1, 1′			116.2, 118.5
2, 2′			125.74, 125.93/125.6125.91
3, 3′	6.52, 6.20	6.48, 6.19	108.1, 106.0
4, 4′			145.9, 147.6
5, 5′			137.6, 138.3
6, 6′			144.9, 145.3
7, 7′			169.4, 169.2/169.3, 169.1
1″			115.1
2″			137.7/137.66
3″			140.82
4″			140.87
5″			143.8
6″	7.06	6.95	109.9
7″			167.5
OMe	3.74	3.75	52.5

^a^ Chemical shifts in δ ppm, coupling constant (*J*) expressed in Hz in parentheses.

**Table 2:** Anti-oxidant and HMGR inhibition effects of organic fractions form the leaves of *F. glaberrima*.

Sample	Antioxidant Activity (IC_50_, μg/mL) ^a^			HMGR Inhibition (IC_50_, μg/mL) ^a^
	DPPH	Superoxide Anion Radical	LPO	
*F. glaberrima*-MeOH	11.1 ± 0.97	18.1 ± 3.89	26.3 ± 2.14	2.86 ± 0.24
CH_2_Cl_2_	38.4 ± 3.10	>50	75.2 ± 2.57	19.9 ± 2.73
EtOAc	4.62 ± 0.28	4.07 ± 0.08	9.67 ± 0.14	1.73 ± 0.23
BuOH	5.25 ± 0.13	4.64 ± 0.23	18.8 ± 0.58	0.74 ± 0.30
*F. glaberrima*-30EtOH	22.1 ± 1.10	11.3 ± 4.73	40.1 ± 4.91	3.67 ± 0.78
70EtOH	19.9 ± 0.45	14.9 ± 0.97	34.7 ± 0.76	3.38 ± 0.55

^a^ IC_50_ data represent mean ± SD of *n* = 3.

**Table 3:** Anti-oxidant and HMGR inhibition effects of isolates from the leaves of *F. glaberrima*.

Compound	Antioxidant Activity (IC_50_, μM) ^a^			HMGR Inhibition (IC_50_, μM) ^a^
	DPPH	Superoxide Anion Radical	LPO	
**1**	3.62 ± 0.57	4.29 ± 0.23	3.54 ± 0.29	1.46 ± 0.22
**2**	3.00 ± 0.08	5.53 ± 0.21	6.02 ± 0.14	17.7 ± 0.04
**3**	4.27 ± 0.45	4.25 ± 0.11	3.83 ± 0.52	8.40 ± 0.84
**4**	3.19 ± 0.13	3.21 ± 0.05	3.75 ± 0.52	12.2 ± 0.50
**5**	4.34 ± 0.17	4.75 ± 0.14	3.61 ± 0.34	26.7 ± 0.90
**6**	4.70 ± 0.14	4.10 ± 0.53	3.93 ± 0.53	>50
**7**	3.81 ± 0.19	4.47 ± 0.07	3.87 ± 0.61	4.98 ± 0.18
**8**	20.9 ± 0.98	3.60 ± 0.23	3.54 ± 0.15	13.8 ± 1.53
**9**	3.94 ± 0.25	4.64 ± 0.45	5.88 ± 0.13	41.5 ± 3.90
**10**	15.7 ± 0.15	21.5 ± 2.03	34.1 ± 2.98	>50
**11**	23.0 ± 0.45	17.7 ± 0.30	>50	>50
**Quercetin**	17.3 ± 1.09	21.3 ± 0.34	7.45 ± 1.33	n.d. ^b^
**Trolox**	31.3 ± 1.27	>50	33.2 ± 3.60	n.d. ^b^
**Resveratrol**	42.1 ± 1.91	>50	47.6 ± 6.35	n.d. ^b^
**Vitamin C**	31.7 ± 0.21	>50	n.d. ^b^	n.d. ^b^
**Pravastatin**	n.d. ^b^	n.d. ^b^	n.d. ^b^	0.41 ± 0.05

^a^ IC_50_ data represent mean ± SD of *n* = 3. ^b^ n.d.: not detected.

## Data Availability

Data will be made available on request.

## Supplementary Materials

The following supporting information can be downloaded at: https://www.mdpi.com/article/10.3390/molecules29020354/s1, Figure S1: HR-ESI-MS spectrum of rugosin B methyl ester (1), Figure S2: ^1^H-NMR spectrum of rugosin B methyl ester (1) in CD_3_OD (400 MHz), Figure S3: ^13^C-NMR spectrum of rugosin B methyl ester (1) in CD_3_OD (100 MHz), Figure S4: ^1^H-^1^H COSY spectrum of rugosin B methyl ester (1), Figure S5: HSQC spectrum of rugosin B methyl ester (1), Figure S6: HMBC spectrum of rugosin B methyl ester (1), Figure S7: Key HMBC correlations for compounds 1 and 20, Figure S8: HR-ESI-MS spectrum of 6′-*O*-galloylrosamultin (20), Figure S9: ^1^H-NMR spectrum of 6′-*O*-galloylrosamultin (20) in CD_3_OD (400 MHz), Figure S10: ^13^C-NMR spectrum of 6′-*O*-galloylrosamultin (20) in CD_3_OD (100 MHz), Figure S11: ^1^H-^1^H COSY spectrum of 6′-*O*-galloylrosamultin (20), Figure S12: HSQC spectrum of 6′-*O*-galloylrosamultin (20), Figure S13: HMBC spectrum of 6′-*O*-galloylrosamultin (20), Figure S14: HPLC chromatograms of the isolated compounds from leaves of Korean meadowsweet (*Filipendula glaberrrima*); Measurement of antioxidant activity: DPPH and superoxide anion radical-scavenging assay and assay for inhibitory effect of lipid peroxidation.
