# Supplementary material for: Antioxidant and Inhibitory Activities of Filipendula glaberrima Leaf Constituents against HMG-CoA Reductase and Macrophage Foam Cell Formation
Source: Molecules. 2024 Jan 10;29(2):354. doi: 10.3390/molecules29020354 (PMC10818259; doi:10.3390/molecules29020354)
Supplement: Supplementary file 1 [file molecules-29-00354-s001.zip › molecules-2774597-supplementary.pdf]

Antioxidant and inhibitory activities of *Filipendula glaberrima* leaf constituents against HMG-CoA reductase and macrophage foam cell formation

You Bin Cho, Hyunbeom Lee, Hui-Jeon Jeon, Jae Yeol Lee*, Hyoung Ja Kim*

* Corresponding authors.

E-mail: ljy@khu.ac.kr (Jae Yeol Lee),

khj@kist.re.kr (Hyoung Ja Kim)

List of Supplementary Materials

Fig. S1. HR-ESI-MS spectrum of rugosin B methyl ester (**1**) 3

Fig. S2. ^{1}H-NMR spectrum of rugosin B methyl ester (**1**) in CD_3OD (400 MHz) 3

Fig. S3. ^{13}C-NMR spectrum of rugosin B methyl ester (**1**) in CD_3OD (100 MHz) 4

Fig. S4. ^{1}H-^{1}H COSY spectrum of rugosin B methyl ester (**1**) 4

Fig. S5. HSQC spectrum of rugosin B methyl ester (**1**) 5

Fig. S6. HMBC spectrum of rugosin B methyl ester (**1**) 5

Fig. S7. Key HMBC correlations for compounds **1** and **20** 6

Fig. S8. HR-ESI-MS spectrum of 6'-*O*-galloylrosamultin (**20**) 6

Fig. S9. ^{1}H-NMR spectrum of 6'-*O*-galloylrosamultin (**20**) in CD_3OD (400 MHz) 7

Fig. S10. ^{13}C-NMR spectrum of 6'-*O*-galloylrosamultin (**20**) in CD_3OD (100 MHz) 7

Fig. S11. ^{1}H-^{1}H COSY spectrum of 6'-*O*-galloylrosamultin (**20**) 8

Fig. S12. HSQC spectrum of 6'-*O*-galloylrosamultin (**20**) 8

Fig. S13. HMBC spectrum of 6'-*O*-galloylrosamultin (**20**) 9

Fig. S14. HPLC chromatograms of the isolated compounds from leaves of Korean meadowsweet (*Filipendula glaberrrima*) 10

Measurement of antioxidant activity

DPPH and superoxide anion radical scavenging assay 11

Assay for inhibitory effect of lipid peroxidation 11

MS Spectrum _ negative mode

sample

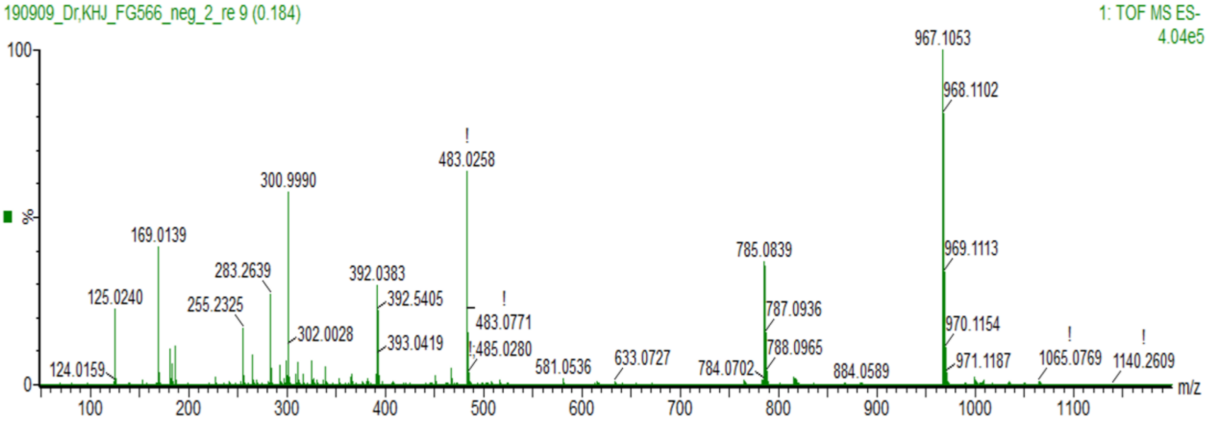

Figure S1. HR-ESI-MS spectrum of rugosin B methyl ester (**1**)

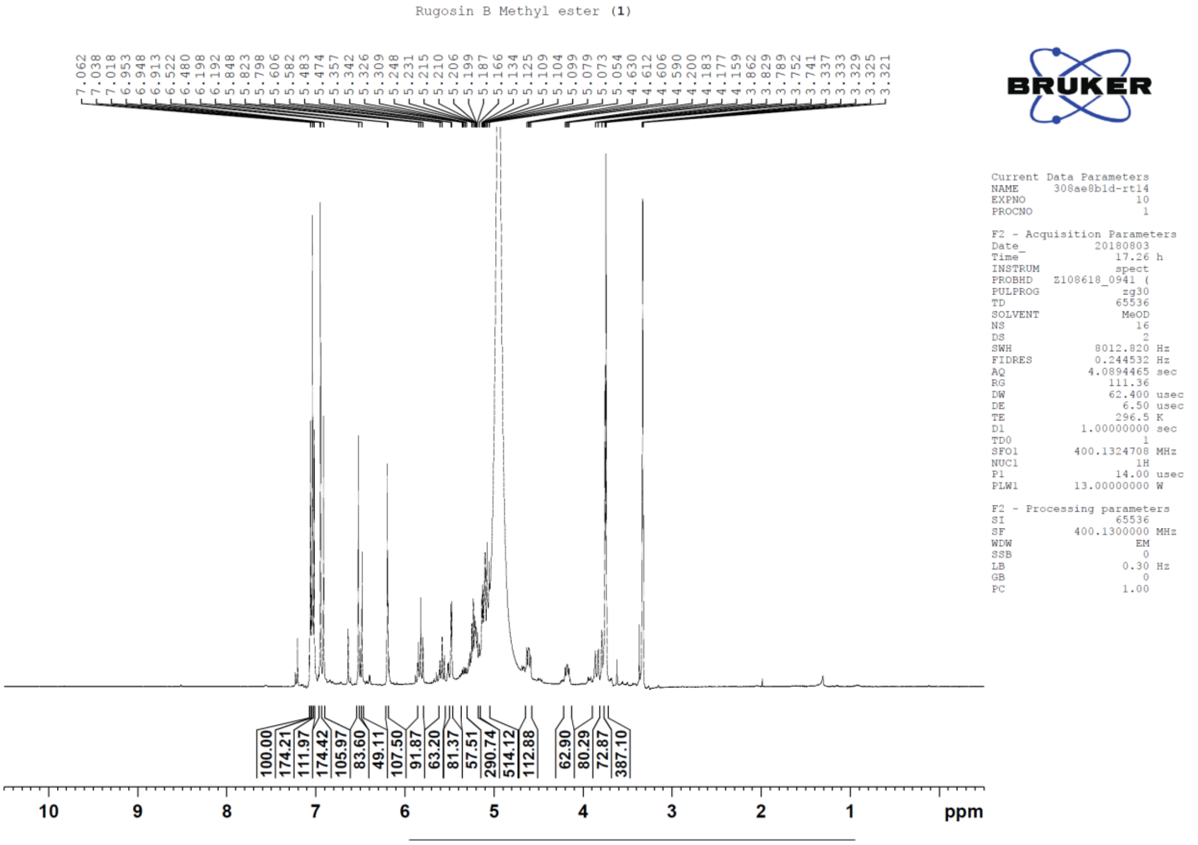

Figure S2. ^{1}H-NMR spectrum of rugosin B methyl ester **(1)** (CD_3OD, 400 MHz).

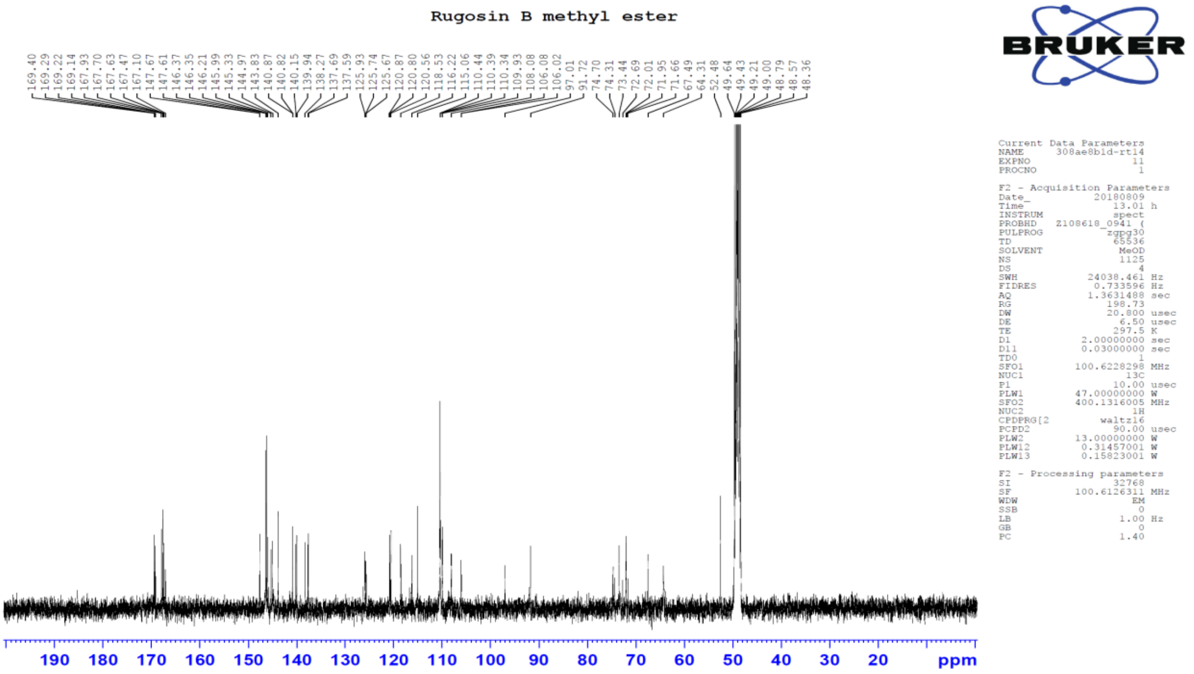

Figure S3. ^{13}C-NMR spectrum of rugosin B methyl ester (**1**) (CD_3OD, 100 MHz).

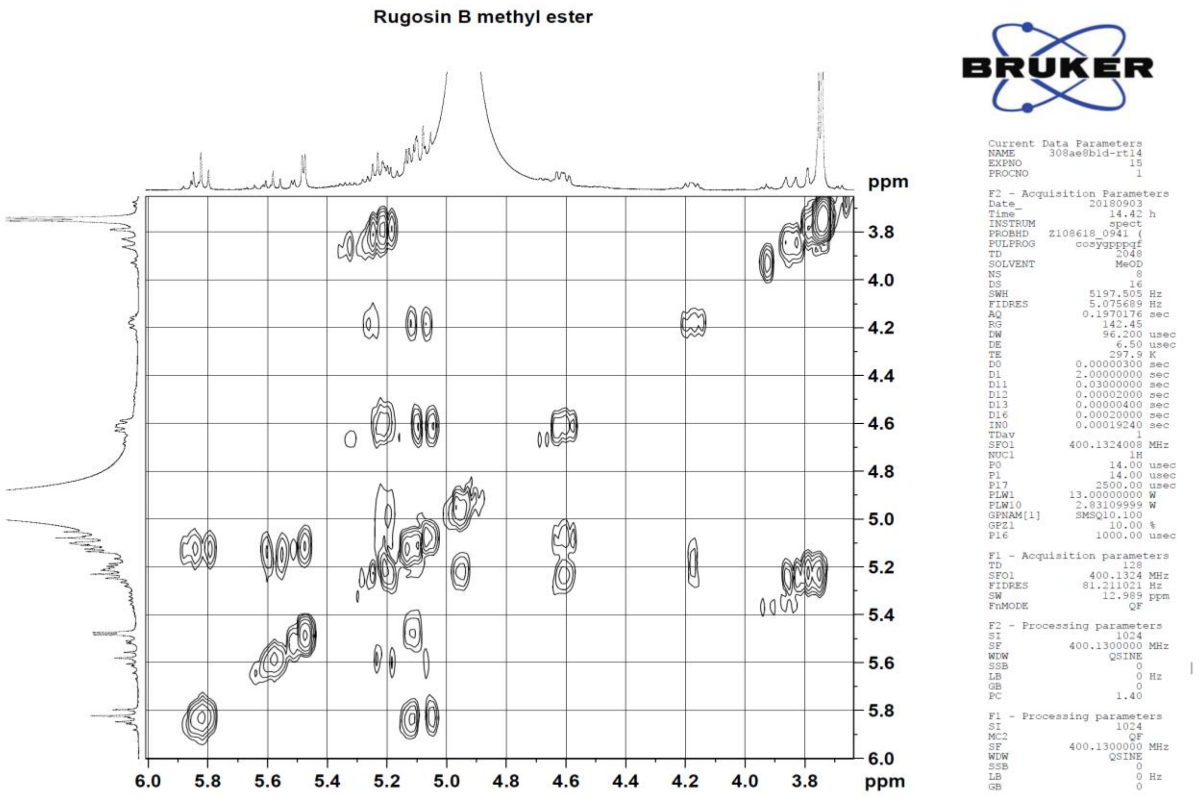

Figure S4. ^{1}H-^{1}H COSY spectrum of rugosin B methyl ester (**1**).

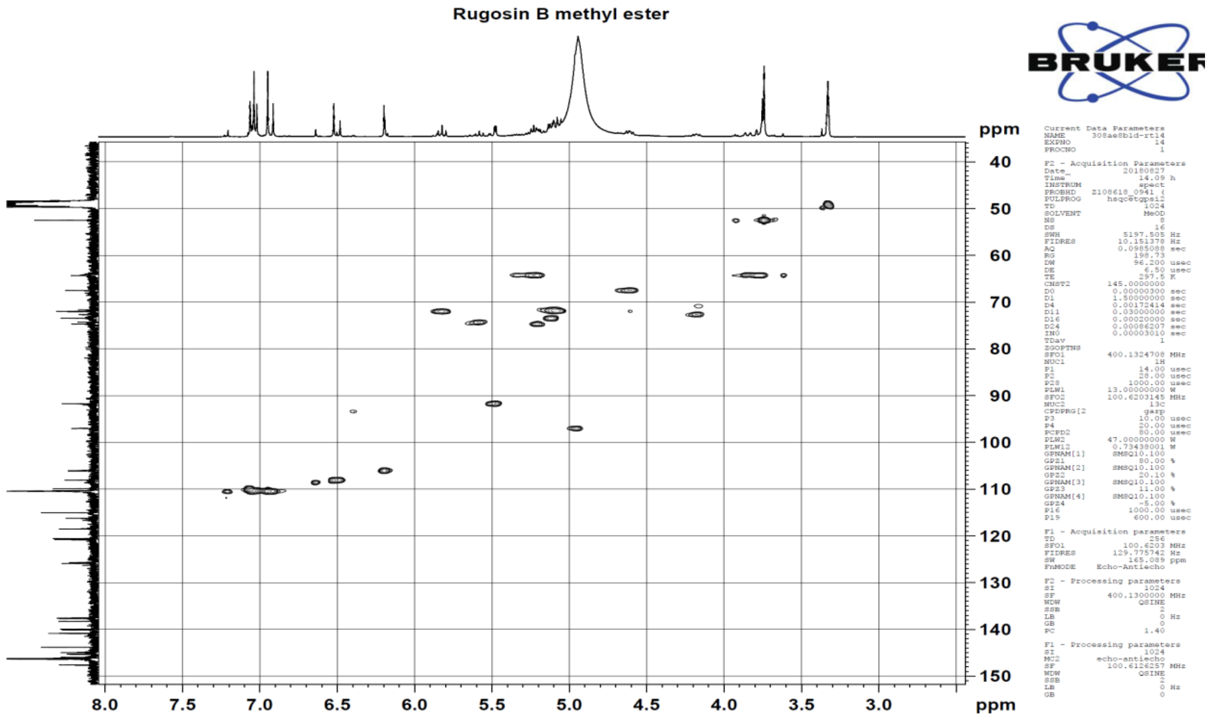

Figure S5. HSQC spectrum of rugosin B methyl ester (**1**).

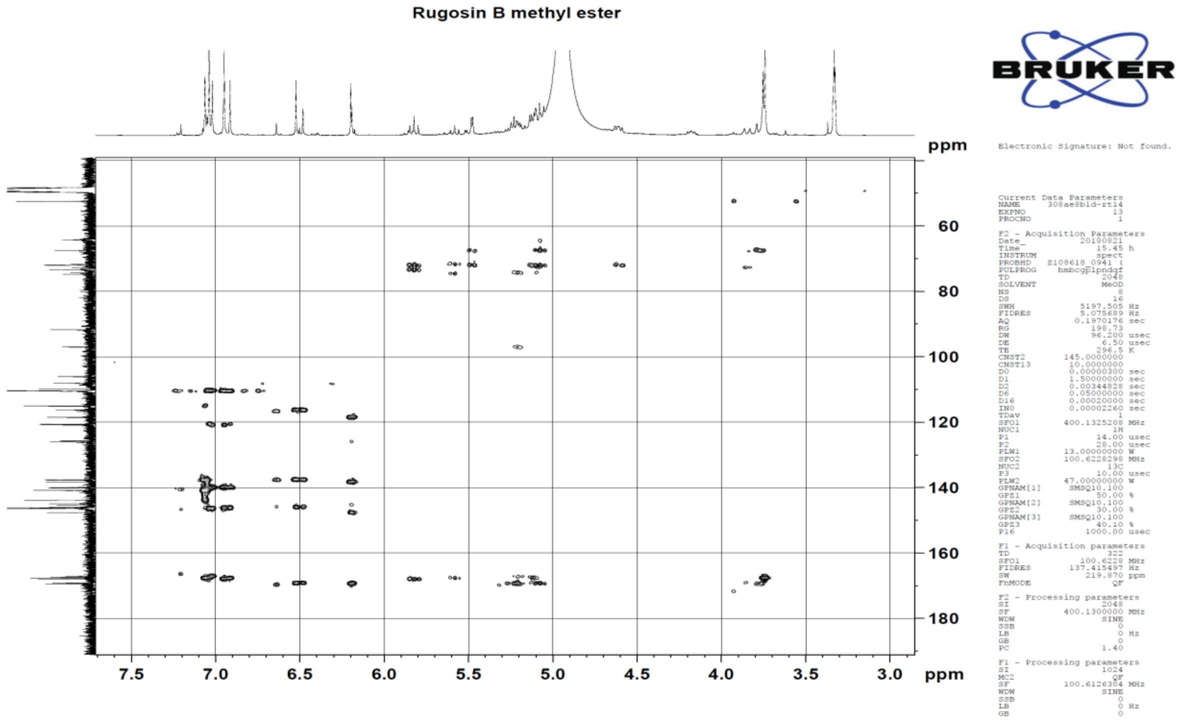

Figure S6. HMBC spectrum of rugosin B methyl ester (**1**).

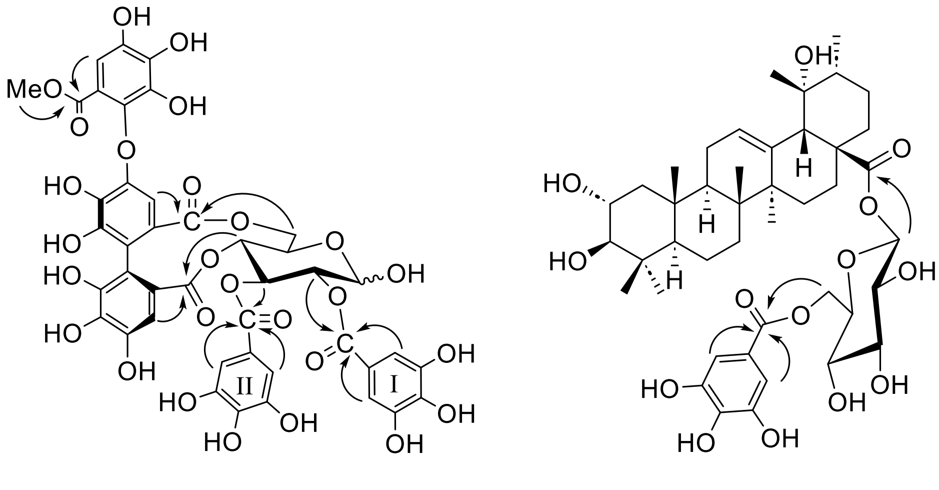

1 **20**

Figure S7. Key HMBC correlations for compounds **1** and **20.**

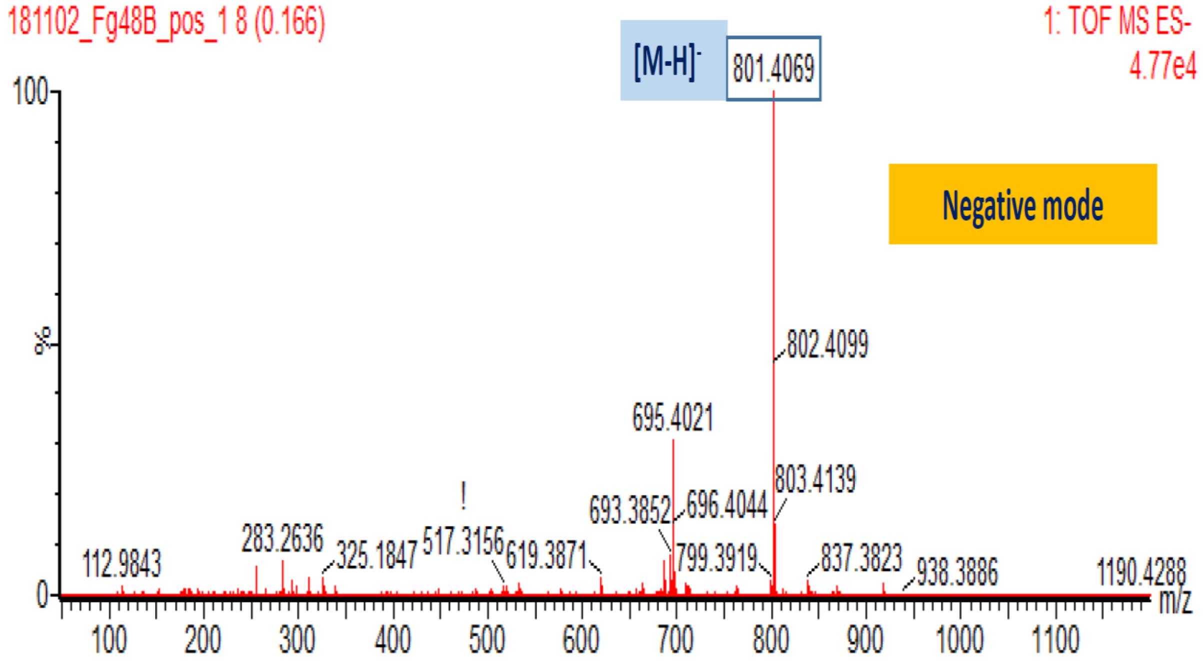

Figure S8. HR-ESI-MS spectrum of 6'-*O*-galloylrosamultin (**20**)

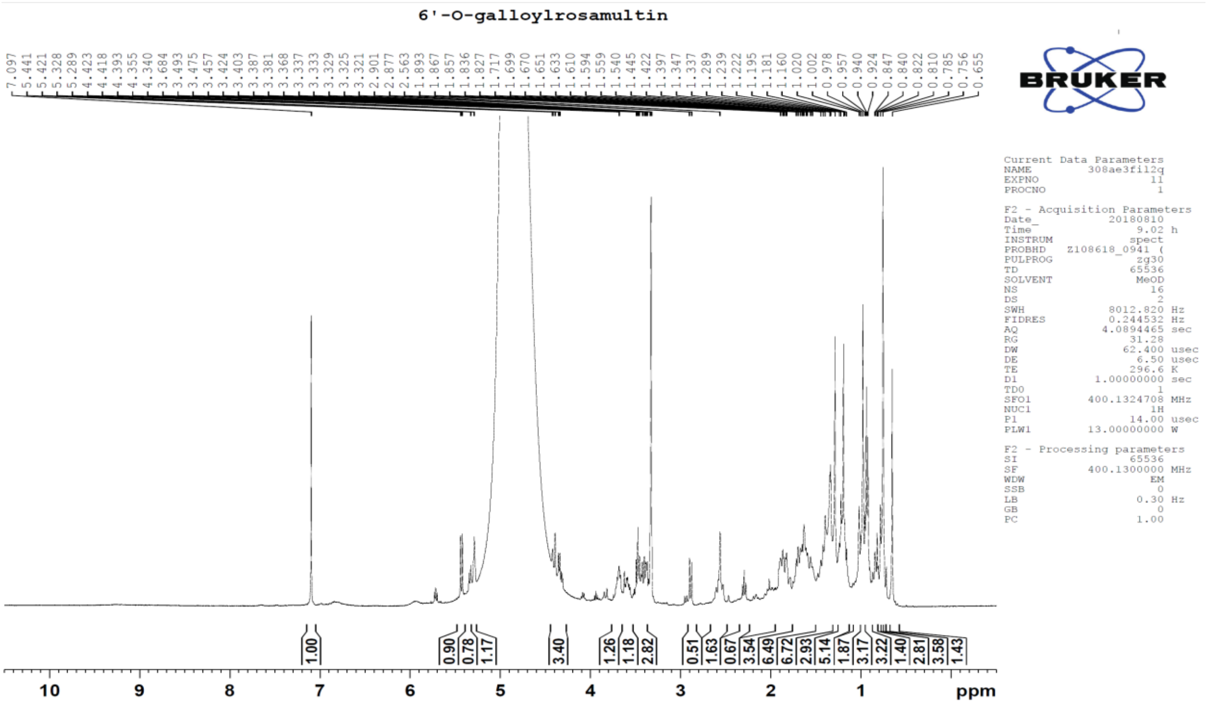

Figure S9. ^{1}H-NMR spectrum of 6'-*O*-galloylrosamultin (**20**) (CD_3OD, 400 MHz).

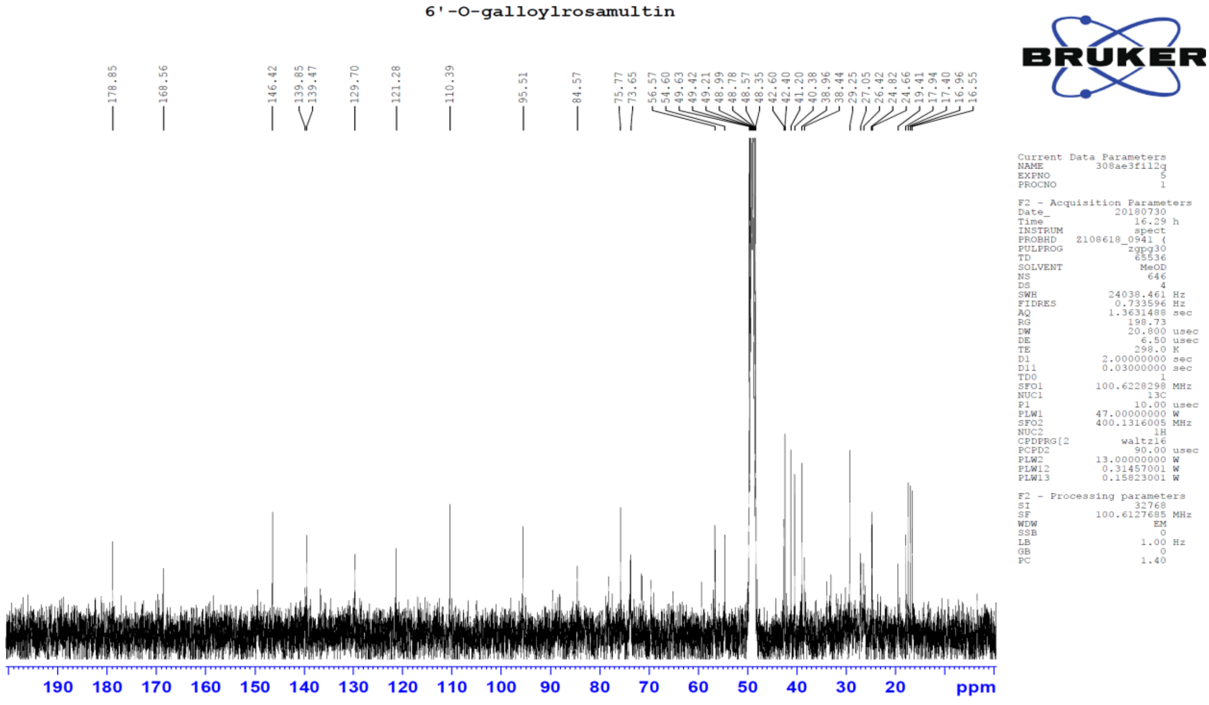

Figure S10. ^{13}C-NMR spectrum of 6'-*O*-galloylrosamultin (**20**) (CD_3OD, 100 MHz).

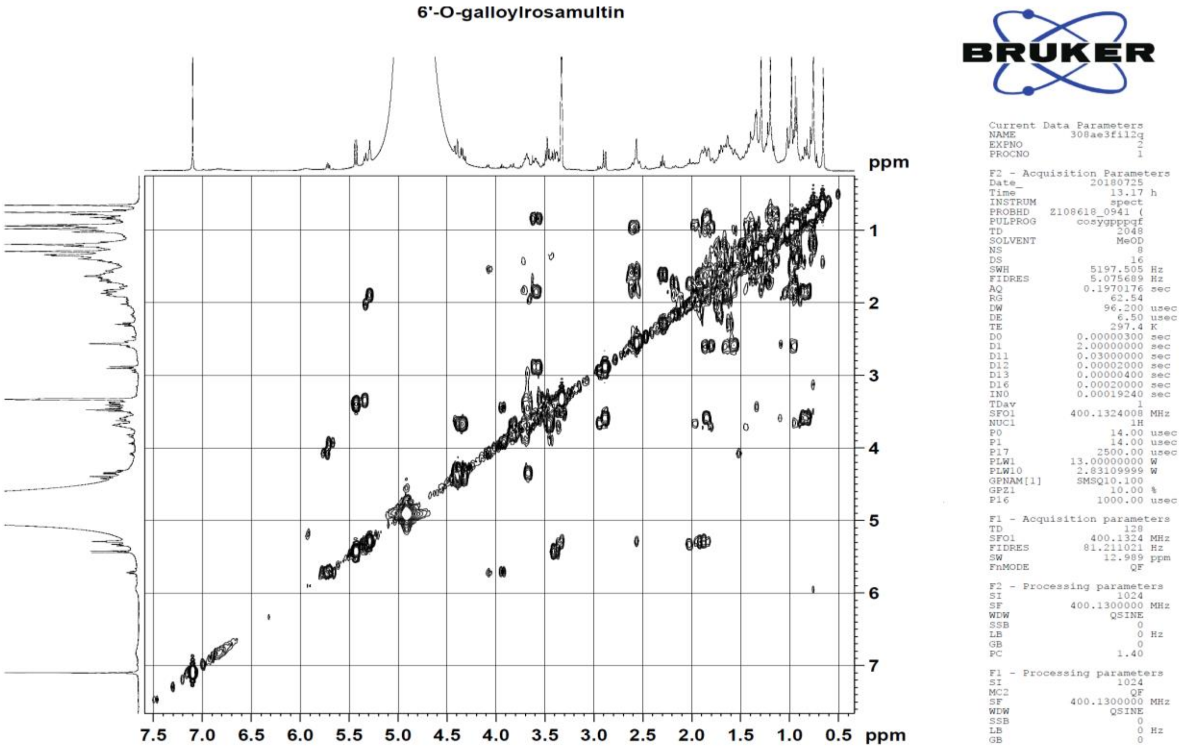

Figure S11. ^{1}H-^{1}H COSY spectrum of 6'-*O*-galloylrosamultin (**20**).

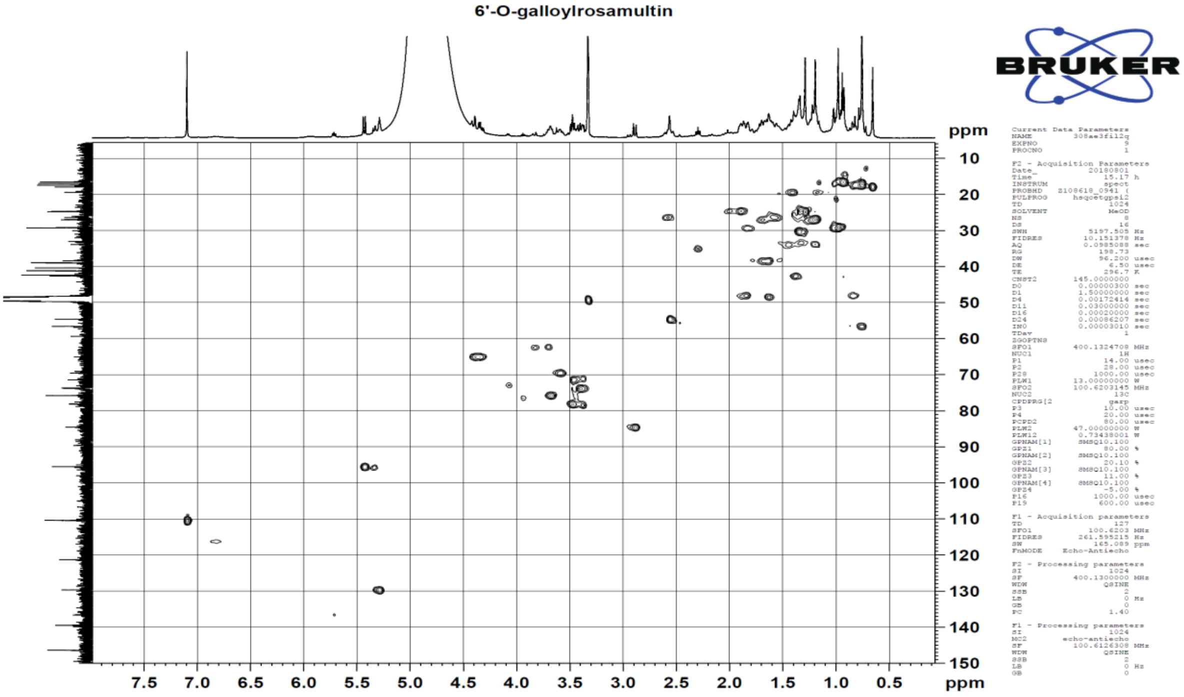

Figure S12. HSQC spectrum of 6'-*O*-galloylrosamultin (**20**).

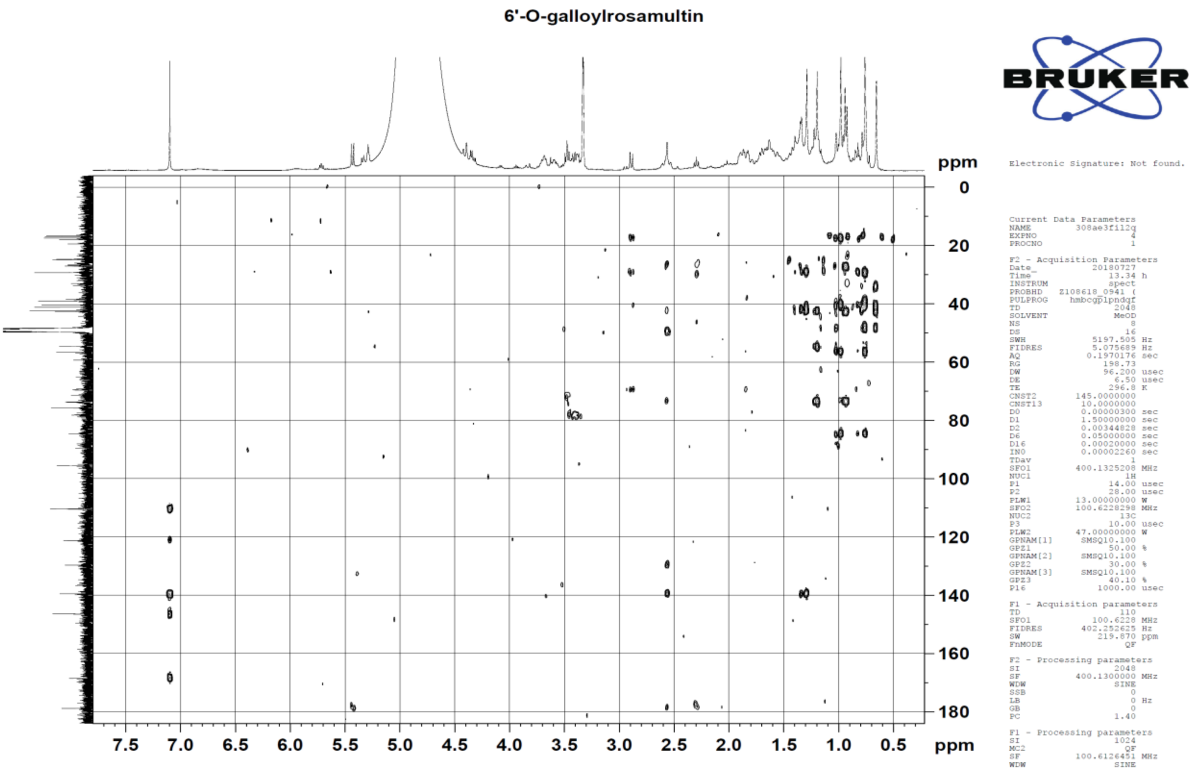

Figure S13. HMBC spectrum of 6'-*O*-galloylrosamultin (**20)**.

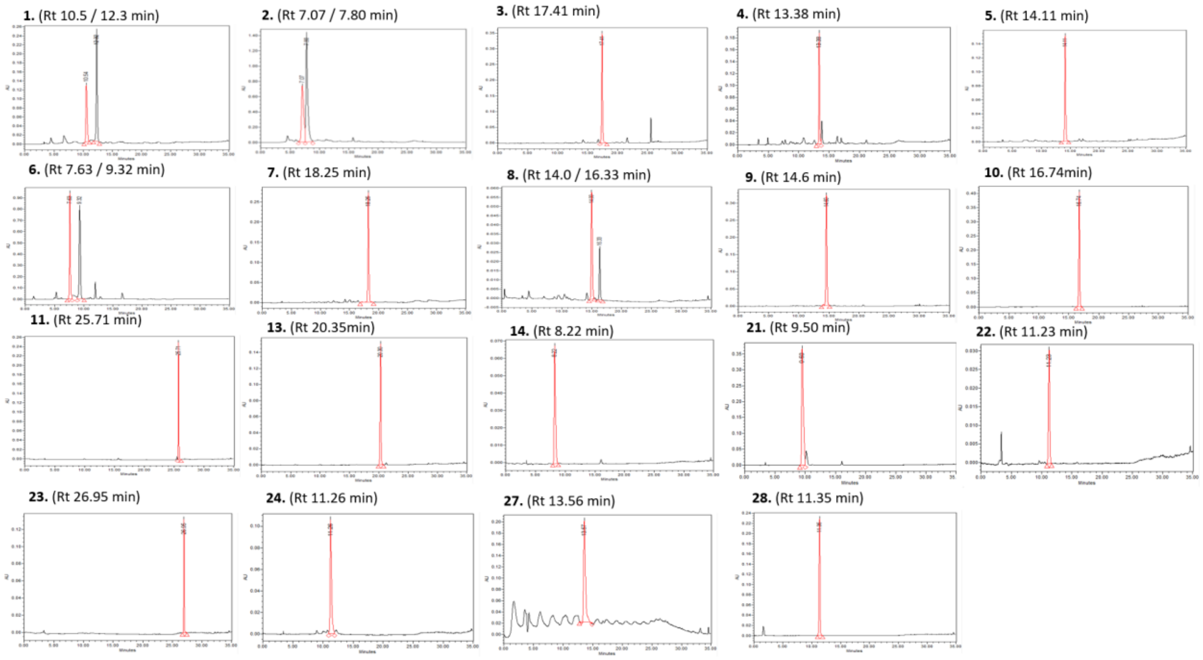

Figure S14. HPLC chromatograms of the isolated compounds from leaves of Korean meadowsweet (*Filipendula glaberrrima*). The HPLC separation was carried out with a Waters 1500 HPLC system equipped with a 1525 binary pump, column oven, and photodiode array detector (model 2996) using a Luna C18 column (5 µm, 250 × 4.6 mm, Phenomenex, Torrance, CA, USA). Solvent A was 0.5% (v/v) phosphoric acid (PPA) in acetonitrile and solvent B was 0.5% (v/v) PPA in water. The linear gradient elution used was as follows: 19–30% A in B over 20 min; 30–100% A in B over 10 min; 19% A in B held for 5 min. The flow rate was 1.0 mL/min. The detection wavelength was 254 nm. **1**: rugosin B methyl ester, **2**: rugosin B, **3**: rugosin A methyl ester, **4**: rugosin A, **5**: eugeniin, **6**: tellimagrandin I, **7:** 1,2,3,4,6-penta-*O*-galloyl-β-D-glucose, **8**: 2,3,4,6-tetragalloyl glucose, **9**: 1,2,3,6-tetra-*O*-galloyl-*β*-D-glucose, **10**: quercetin 3-glucuronic acid, **11**: kaempferol 8-*O*-glucuronic acid, **13**: kaempferol 3-glucuronic acid, **14**: catechin, **21**: methylgallate, **22**: methylprotocatechuate, **23**: salicylic acid, **24**: gaultherin, **27**: salidroside, **28**: undulatoside A.

DPPH radical scavenging assay.

The extracts and isolates were dissolved in dimethyl sulfoxide (DMSO) at concentrations ranging from 0.1 to 10 mg/mL and 0.1 to 10 mM, respectively. Subsequently, they were combined with an ethanol solution of DPPH (100 μM). The DPPH solution (190 μL per well) and the samples (10 μL per well) were incubated in a 96-well microplate at 37 °C for 30 minutes under dark conditions. The absorbance at 515 nm was quantified using a microplate reader, and the percentage inhibition (%) of each sample was calculated by comparing it with the control group treated with DMSO. Linear regression analysis was employed to estimate the IC_{50} values, which indicated the concentration of test samples required to inhibit 50% of the free radicals.

Superoxide anion radical scavenging assay.

Test compounds or DMSO blanks were incubated for 30 min at 37 °C in 0.1 M potassium phosphate buffer (pH 7.5) containing 0.1 mM EDTA, 0.1 mM NBT, 0.3 mM xanthine, and 0.02 U/mL xanthine oxidase (EC 1.2.3.2). The reaction was concluded by introducing 1 N HCl, and the absorbance of the resulting formazan was measured at 540 nm. The absorbance of the formazan was quantified at 540 nm, and the results are presented as the mean of triplicate measurements.

Assay for inhibitory effect of lipid peroxidation. Male Sprague Dawley rats were used in the experiment, and only water was supplied for 24 h before the experiment. The study was carried out following the guidelines for the handling and utilization of laboratory animals and received approval from the Animal Research Ethics Committee of the Korea Institute of Science and Technology (approval number: KISTIACUC-2018-081). Experimental animals were

anesthetized with isoflurane and dissected. A 0.15 M ice-cold KCl solution was perfused through the liver portal vein to remove blood from the liver and extract the liver. The liver was homogenized in KCl solution at 10 times the liver's weight, and the protein concentration was quantified by the Bradford protein method using bovine serum albumin as a standard. A lipid peroxidation test was performed using a slightly modified version of the method described. Reaction mixtures consisted of 300 μL of liver homogenate (11 mg protein/mL), 300 μL of 50 mM Tris-HCl buffer (pH 7.5) containing 10 μM $FeSO_4$, and 0.4 mM ascorbic acid. Test samples (10 μL) or DMSO blanks were added to the reaction mixture and incubated at 37 °C for 30 min. After incubation, lipid peroxidation was measured by forming thiobarbituric acid reactive substances. The mixture was subjected to a reaction at 90 °C for 30 min, followed by cooling and subsequent centrifugation (5,000 × g) for 10 min. The absorbance of the resulting supernatant was determined at 535 nm, and the percentage of lipid peroxidation inhibition by the sample was computed. DMSO was utilized as a control instead of the test drug, and the concentration (IC_{50}) of the sample necessary to hinder the formation of lipid peroxide by 50% was assessed.
